# Aloe-emodin Attenuates *Staphylococcus aureus* Pathogenicity by Interfering With the Oligomerization of α-Toxin

**DOI:** 10.3389/fcimb.2019.00157

**Published:** 2019-05-15

**Authors:** Lanxiang Jiang, Tian Yi, Ziying Shen, Zihao Teng, Jianfeng Wang

**Affiliations:** ^1^Department of Dermatology, Second Hospital of Jilin University, Jilin University, Changchun, China; ^2^Key Laboratory of Zoonosis Research, Ministry of Education, Institute of Zoonosis, College of Animal Science, Jilin University, Changchun, China; ^3^Laboratory Animal Center, College of Animal Sciences, Jilin University, Changchun, China

**Keywords:** *Staphylococcus aureus*, α-toxin, aloe-emodin, antibiotic-resistant, pneumonia

## Abstract

α-toxin, an essential virulence factor secreted by *Staphylococcus aureus* (*S. aureus*), is a critical exotoxin in multiple infections. In this study, we found that aloe-emodin (AE), a natural compound lacking anti-*S. aureus* activity, could inhibit the hemolytic activity of α-toxin. Oligomerization assays, molecular dynamics simulations, and fluorescence-quenching analyses were used to determine the mechanism of this inhibition. The oligomerization of α-toxin was restricted by the engagement of AE with K110, T112, and M113 of the toxin, which eventually resulted in inhibition of the hemolytic activity. Lactate dehydrogenase and live/dead assays demonstrated that AE decreased the injury of human lung epithelial cells (A549) and mouse lung macrophages (MH-S) mediated by *S. aureus*. Furthermore, treatment with AE showed robust protective effects in mice infected by *S. aureus*. These findings suggest that AE effectively inhibited the pore-forming activity of α-toxin and showed a protective effect against *S. aureus* virulence *in vitro* and *in vivo*, which may provide a new strategy and new antibacterial agent for clinical treatment of *S. aureus* infections.

## Introduction

*Staphylococcus aureus* (*S. aureus*), one of the most common pathogens, is a critical cause of many local and systemic infections ranging from pneumonia, sepsis, and andocarditis, to osteomyelitis. Among these diseases, *S. aureus* pneumonia is one of the most serious ventilator-associated infections, with ~10–25% mortality and, for some secondary infections, the rate can even reach 75% (Gillet et al., 2002; Del Giudice et al., 2011; Li et al., 2011; Chastre et al., 2014). Although lactams, aminoglycosides, tetracyclines, sulfonamides, and other major antimicrobial drugs have been commonly used in the past century, we still cannot effectively inhibit the *S. aureus* pneumonia observed in the clinic most of the time. However, the abuse of antibiotics has led to many resistant strains, and treatment of *S. aureus* infections has cost at least 450 million dollars due to the increasing resistance (Parvizi et al., 2010; Song et al., 2010). In Europe, ~10–25% of *S. aureus* isolated from hospitals were observed to be methicillin-resistant *S. aureus* (MRSA), and the proportion has reached 50% in some regions (Commun, 2011). Even worse, the proportion of MRSA appeared to reach the highest level in years in parts of east Asia, such as Taiwan and South Korea, with an average rate of 77.6% (Chen and Huang, 2014). Since the twentieth century, the multiresistance of MRSA has become more complicated, which typically results in a delay in clinical treatment (Mendes et al., 2013). Currently, vancomycin is the most commonly used drug to treat MRSA-associated pneumonia (Wunderink et al., 2003). However, the sensitivity of MRSA to vancomycin has been gradually decreasing for years and, given the current trends, the time required for the spread of resistant strains is much less than the time required for research and application of a new medicine. Accordingly, no treatments may be available for MRSA pneumonia in the future, and we need a new treatment strategy to replace the old antibiotic use regimens. Several studies have reported that targeting virulence factors typically results in weak pathogenicity of pathogens, suggesting that this may be a promising strategy in the treatment of *S. aureus* pneumonia (Qiu et al., 2012a,b; Wang et al., 2016).

During infection, a variety of virulence factors are secreted for invasion and colonization, including exotoxin and surface-associated protein (Vandenesch et al., 2012). α-toxin is one of the most important exotoxins produced by *S. aureus* and plays a key role in the course of multiple diseases as a pore-forming protein. It is a 33.2 kDa water-soluble monomer encoded by *hla* and can oligomerize into a 232.4 kDa membrane-inserted heptamer that penetrates the membrane (Gouaux, 1998; Nguyen and Kamio, 2004). The oligomer comprises seven monomers and consists of three major domains, including the cap domain, the rim domain, and the stem domain, which forms the transmembrane channel (Gouaux et al., 1994; Song et al., 1996). Many types of mammalian cells, including monocytes, erythrocytes, macrophages, and epithelial cells, are sensitive to α-toxin (Gouaux, 1998; Nygaard et al., 2012). For *S. aureus* pneumonia, studies have reported the destructive effect of α-toxin on the air-blood barrier, and a mutant strain lacking α-toxin showed decreased toxicity in animal models (McElroy et al., 1999; Xu et al., 2015). Therefore, targeting α-toxin is a promising therapeutic strategy for *S. aureus* infections, particularly MRSA pneumonia.

**Figure 1:**
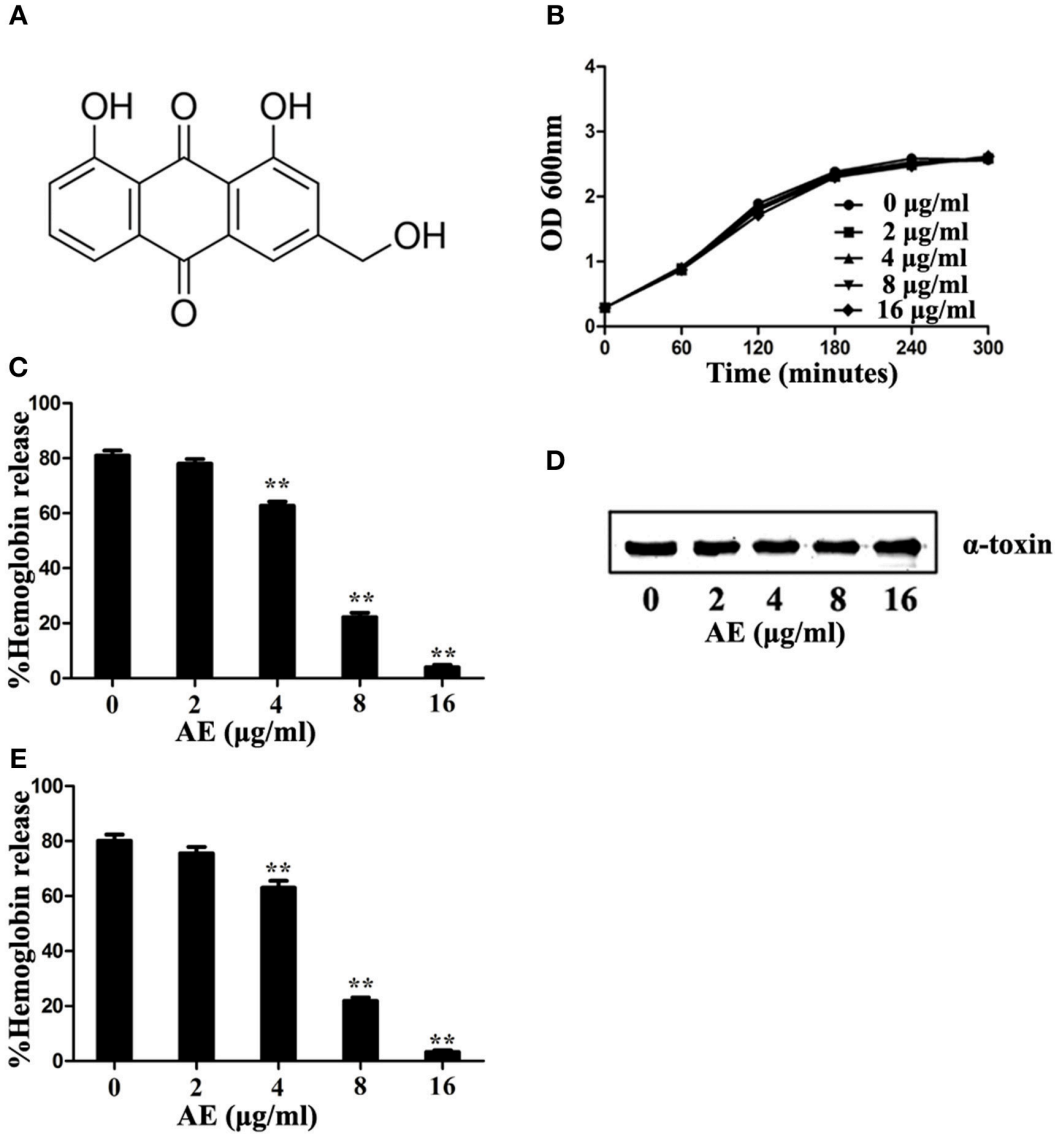
Aloe-emodin (AE) inhibits the hemolytic activity of α-toxin. **(A)** Chemical structure of AE. **(B)** Growth curves of USA300 treated with the indicated concentrations of AE. The OD_600_ values of each sample were monitored every 60 min. **(C)** USA300 was cultured with various concentrations of AE and, then, the supernatants were used for the determination of hemolytic activity. The hemolytic activity of culture supernatant of *S. aureus* co-cultued with AE was inhibited, with activities of 80.97, 78.00, 62.75, 22.19, and 4.00% observed in supernatants containing 0, 2, 4, 8, and 16 μg/ml AE, respectively. **(D)** USA300 was cultured with various concentrations of AE and the expression of α-toxin in the culture supernatant demonstrated by Western blot. **(E)** Hemolytic activity of purified α-toxin treated with or without AE. Bars represent the mean values of the experiments. The AE treatment reduced the observed hemolytic activity from 80.15% (0 μg/ml) to 75.51, 63.06, 22.88, and 3.26% when supernatants contained 2, 4, 8, and 16 μg/ml AE, respectively (^**^ indicates *P* < 0.01 compared with the AE-free group; two-tailed Student's *t*-test).

Aloe-emodin [AE; 1,8-dihydroxy-3-(hydroxymethyl)-anthraquinone] (Figure 1A) is a common active compound derived from the leaves of *Aloe vera* and *Rheum officinale* (Dutta et al., 2007) that has been reported to possess antimicrobial, antiviral, and hepatoprotective activities (Eshun and He, 2004) as well as anticancer activity toward hepatoma cells, lung squamous cell carcinoma, and neuroectodemal tumors (Pecere et al., 2000; Lee, 2001; Kuo et al., 2002). In this study, we observed that AE can inhibit the hemolytic activity of *S. aureus* without decreasing the expression of α-toxin. In addition, we evaluated the protective effect of AE against MRSA *in vitro* and *in vivo*.

## Materials and Methods

### Bacterial Strain and Reagents

The MRSA strain USA300 (BAA-1717) used in this study was purchased from the American Type Culture Collection (ATCC). AE was purchased from Chengdu Herbpurify Co., Ltd. (purity > 98%) (Chengdu, China) and was prepared in DMSO (Sigma-Aldrich). The bacteria were cultured in tryptic soy broth (TSB, Qingdao Hope Biol-Technology Co., Ltd.) at 37°C.

### MIC Determination

The MIC of AE against USA300 was assessed via the broth microdilution method according to Clinical and Laboratory Standards Institute guidelines. The MIC value was defined as the lowest concentration of AE at which no visible growth of the microorganism could be observed.

### Growth Curve Assay

The USA300 strain was grown in TSB with shaking at 200 rpm at 37°C to an OD value of 0.3 at 600 nm, after which the cultures were aliquoted into five Erlenmeyer flasks. Various doses of AE were added into four of the cultures at concentrations of 2, 4, 8, and 16 μg/ml, maintaining the final concentration of DMSO in all the cultures at 1% (v/v). The control cultures were treated with 1% DMSO. Following the addition of AE, the bacteria were grown at 37°C with shaking at 200 rpm, and the OD_600_ values of the cultures were monitored every 60 min.

### Hemolysis Assay

Hemolysis assays were used to determine the inhibitory effect of AE on the hemolytic activity of α-toxin. The USA300 supernatants were harvested by centrifugation at 10,000 rpm for 1 min at 4°C, and then 0.1 ml of each supernatant was added to 1 ml of PBS buffer. The mixture was preincubated with AE at concentrations of 2, 4, 8, and 16 μg/ml at 37°C for 20 min. Next, 25 μl of rabbit erythrocytes (5 × 10^6^ cells/ml) was added to each tube. Following an additional 30 min incubation at 37°C, the unlysed erythrocytes were removed by centrifugation at 6,000 rpm for 1 min, and the hemolytic activity was assessed by determining the OD_543_ values of the supernatant. For the control group, 25 μl of rabbit erythrocytes was added to 975 μl distilled water, and the supernatant served as the 100% hemolysis control. The % hemolysis of the AE-treated samples were calculated by comparing the OD_543_ values of the samples with that of the control cultures.

We also compared the effect of AE on the wild-type and mutant forms of α-toxin. Purified proteins (100 ng/ml) were preincubated with various concentrations of AE at 37°C for 20 min. Next, rabbit erythrocytes were added to the mixtures and incubated for another 30 min at 37°C. Erythrocyte lysis was determined as described above.

### Western Blot Assay

For the Western blot assay, USA300 was grown in TSB at 37°C with shaking at 200 rpm to an OD_600_ of 0.3. Next, the cultures were equally divided into five 50-ml Erlenmeyer flasks with or without AE at concentrations of 2, 4, 8, and 16 μg/ml and then were cultured with shaking at 37°C until reaching the postexponential growth phase (OD_600_ of 2.5). Subsequently, the bacterial cultures were centrifuged at 10,000 rpm for 1 min, and the supernatants were boiled in Laemmli sample buffer and loaded on a 12% sodium dodecyl sulfate-polyacrylamide gel. The proteins were then transferred to polyvinylidene fluoride (PVDF) membranes (Wako Pure Chemical Industries, Ltd., Osaka, Japan). The membranes were blocked with 5% bovine serum albumin (Wako) in PBS at 4°C overnight. Next, a rabbit antibody against α-toxin (diluted to 1:8,000; Sigma-Aldrich) was incubated with membranes at 37°C for 2 h, followed by an incubation with a horseradish peroxidase-conjugated secondary antibody (diluted to 1:4,000; Sigma-Aldrich) under the same conditions. The blots were detected with Amersham ECL Western blotting detection reagents (GE Healthcare, Buckinghamshire, UK) and visualized on Tanon-5200 Chemiluminescent Imaging System (Tanon Science & Technology Co., Ltd., Shanghai, People's Republic of China).

### Deoxycholate-Induced Oligomerization Assay

To assess the deoxycholate-induced oligomerization of α-toxin, 500 ng of purified α-toxin was added to 5 mM deoxycholate and incubated with AE at concentrations of 2, 4, 8, and 16 μg/ml at 22°C for 20 min. Then, the samples were placed in Laemmli sample buffer without β-mercaptoethanol at 55°C for 10 min, and the effect of AE on protein oligomerization was assessed by Western blot assays as described above.

### Molecular Modeling

The initial structure of α-toxin was obtained from the 3D X-ray structure (PDB code: 4YHD). To obtain the starting structure of the AE/α-toxin complex for MD simulation, we used a standard docking procedure for a rigid protein and a flexible ligand using AutoDock 4 (Morris et al., 2009; Hu et al., 2010). Subsequently, the MD simulation of the complex systems was performed, and the detailed processes of the computational analyses were described in a previous report (Dong et al., 2013; Niu et al., 2013).

### Binding Affinity Determination of Ligands With Proteins

To investigate the importance of Lys110, Tyr112, and Met113 in the binding of α-toxin and AE, we mutated these amino acids to alanine. Next, the WT α-toxin and its variants (K110A, Y112A, and M113A mutant proteins) were expressed and purified as described by Qiu et al. (2012a), with the primers used for mutagenesis listed in Table 1. The fluorescence-quenching method was used to measure the binding constants (*K*_*A*_) of AE with the proteins. A 280-nm excitation wavelength with a 5-nm bandpass and a 345-nm emission wavelength with a 10-nm bandpass were used for the measurements. Details of performing the measurements were described previously (Bandyopadhyay et al., 2002; Jurasekova et al., 2009).

**Table 1:** Primers used in this study.

**Primer**	**Oligonucleotide primer sequence (5^**′**^-3^**′**^)**
WT-F	CGCGGATCCGCAGATTCTGATATTAATATTAAAAC
WT-R	CCGCTCGAGTTAATTTGTCATTTCTTCTTTTTC
K110A-F	CCAAGAAATTCGATTGATACAGCGGAGTATATGAGTACTTTAAC
K110A-R	GTTAAAGTACTCATATACTCCGCTGTATCAATCGAATTTCTTGG
T112A-F	GATTGATACAAAAGAGGCTATGAGTACTTTAAC
T112A-R	GTTAAAGTACTCATAGCCTCTTTTGTATCAATC
M113A-F	GATTGATACAAAAGAGTATGCGAGTACTTTAACTTATGG
M113A-R	CCATAAGTTAAAGTACTCGCATACTCTTTTGTATCAATC

### Live/dead Assays and LDH Release

A549 human lung epithelial cells (ATCC CCL-185) and mouse lung macrophages (MH-S, ATCC CRL-2019) were plated in 96-well plates at a density of 2.0 × 10^4^ cells/well and were cultured overnight. The cells were subsequently treated with 200 μl of bacteria at an MOI of 500 with or without AE and then were incubated for 5 h at 37°C. For the live/dead assays, the cells were stained using a live/dead (green/red) reagent (Invitrogen) and detected with a confocal laser scanning microscope (Nikon, Tokyo, Japan). For the LDH assay, the supernatants of the wells were assessed with a Cytotoxicity Detection Kit (LDH, Roche, Basel, Switzerland).

### Animal Infection

Six-to-eight-week-old C57BL/6J female mice were purchased from Jilin University Experimental Animal Center. Animal experiments were approved by and conducted in accordance with the guidelines of the Animal Care and Use Committee of Jilin University. This study was approved by the Animal Welfare and Research Ethics Committee of Jilin University.

USA300 was cultured in TSB with shaking at 200 rpm at 37°C to an OD_600_ of 0.8, and the cells were then harvested by centrifugation for 10 min at 3,000 rpm. The bacteria were resuspended in physiological saline and quantified at OD_600_. The mice were anesthetized with ether and then were administered nasal drops with 20 μl of suspended bacteria (1 × 10^10^ CFUs/mL). The mice in the treatment group were subsequently injected with 100 mg/kg AE by hypodermic injection 2 h after infection and were continually treated for 72 h at 8-h intervals. The mice in the control group were treated with DMSO at the same dosage. Each experimental group in this study contained 10 mice.

The mortality rate of the mice was determined daily. The mouse lungs were immersed in 4% formalin, embedded in paraffin, stained with hematoxylin and eosin, and visualized with a light microscope. For colony count analyses, the lungs were accurately weighed and ground in PBS containing 2% Triton. Finally, the tissue homogenates were plated to quantify the colonization of USA300 in the lungs.

### Statistical Analysis

The statistical analyses between the treated and control group were assessed using SPSS 13.0 software. The log-rank test and Student's *t*-tests were performed for the survival curves and others assays, respectively. ^*^P < 0.05 and ^**^P < 0.01.

## Results

### AE has no Effect on *S. aureus* Growth

The minimum inhibitory concentration (MIC) determination and growth curve assays were performed to determine the antibacterial activity of AE for *S. aureus*. The MIC of AE for the *S. aureus* strain USA300 was >1,024 μg/ml, suggesting that this compound, as a therapeutic agent, lacks antibacterial activity against USA300. Furthermore, USA300 grown with 2–16 μg/ml of AE showed no difference from the control group cultures without AE (Figure 1B).

### AE Inhibits the Hemolytic Activity of α-toxin

Then, the potential inhibitory effect of AE against α-toxin activity was evaluated at the concentrations without antibacterial activity against *S. aureus*. The results of hemolysis assays showed that AE notably inhibited the hemolytic activity of the *S. aureus* supernatant in a dose-dependent manner when cocultured with bacteria. Consistent with previous studies, 80.97% of rabbit erythrocytes were lysed by α-toxin in the control group without AE, and the activity was significantly decreased (to 22.19%) when cultured with 8 μg/ml of AE. Furthermore, only 4.00% of erythrocytes were lysed in the supernatants treated with 16 μg/ml of AE (Figure 1C). The 50% inhibitory concentration (IC_50_) of AE determined to be 4.81 μg/ml.

Western blot assays were used to further determine whether the decreased hemolytic activity was due to a reduction in α-toxin. However, the secretion of α-toxin was barely affected in the supernatants after treatment with various concentrations of AE (Figure 1D). Therefore, we hypothesized that AE may act on α-toxin directly to inhibit its hemolytic activity. To validate this, we used recombinant purified α-toxin in hemolysis assays. Consistent with the hemolysis described above, the hemolytic activity of purified α-toxin was significantly inhibited in the presence of AE (Figure 1E). Taken together, our results suggested that AE inhibits the hemolytic activity of α-toxin by direct interaction with the toxin.

### AE Inhibits the Oligomerization of α-toxin

**Figure 2:**
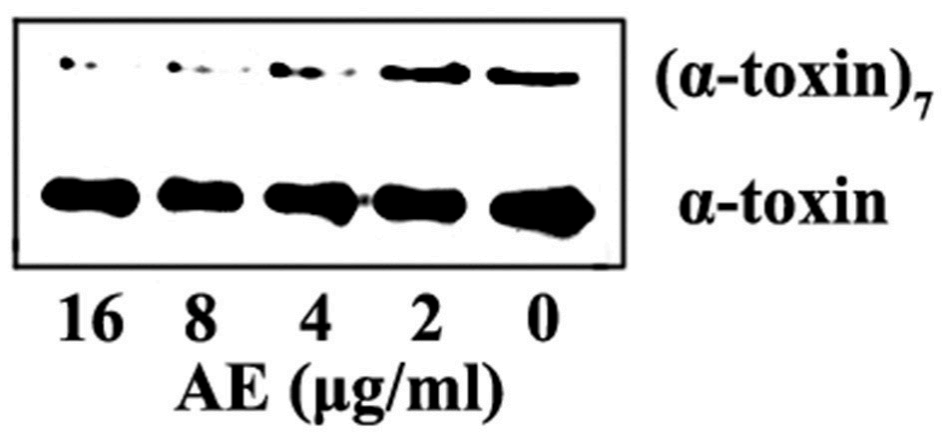
AE interferes with the oligomerization of α-toxin. α-toxin was incubated with 5 mM deoxycholate with various concentrations of AE (0, 2, 4, 8, and 16 μg/ml) at 22°C for 20 min. The oligomerization of toxin was evaluated via Western blot assays.

Deoxycholate-induced oligomerization assays were used to determine whether the loss of hemolytic activity was due to the inhibition of oligomerization, which is critical for the hemolytic activity of α-toxin. As expected, formation of the α-toxin heptamer was significantly inhibited in the sample treated with 4 μg/ml of AE, and the oligomerization was almost completely inhibited when 16 μg/ml of AE was added to the reaction system (Figure 2). Taken together, these results indicate that the loss of hemolytic activity may be due to AE-mediated inhibition of the oligomerization of α-toxin.

### Molecular Dynamics Simulation for α-toxin-AE

**Figure 3:**
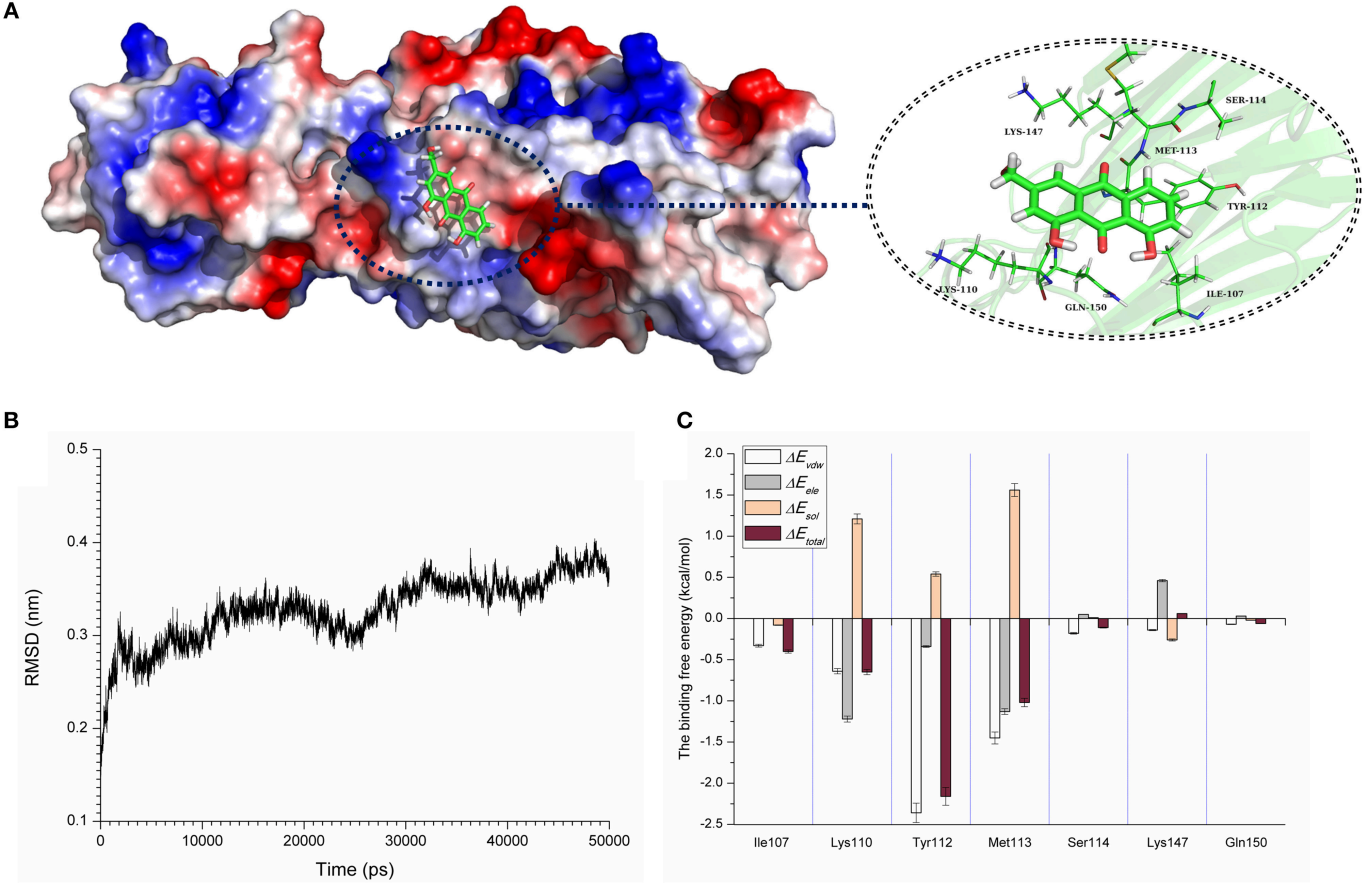
The 3D structure determination of the α-toxin with AE via molecular modeling method by using Gromacs 5.1.0 software. **(A)** The 3D structure of α-toxin binding with AE obtained via molecular modeling. **(B)** The root-mean-square deviations (RMSDs) displayed by the backbone atoms of the protein during MD simulations of α-toxin-AE is presented by using Gromacs 5.1.0 software. The RMSDs as a function of the molecular dynamics simulation time. **(C)** Decomposition of the binding energy on a per-residue basis in the binding sites of the α-toxin-AE complex.

Computational biology assays were further performed to characterize the detailed mechanisms of this inhibition. First, computational biology analyses were used to explore the potential binding mode of AE with α-toxin in the active site. AE binds to α-toxin, and the binding mode is shown in Figure 3A. AE can bind to α-toxin via hydrogen bonding and hydrophobic interactions. During the time course of the simulation, AE could localize to the “stem” region of α-toxin (residue 100 to 150). In detail, the binding model of AE with α-toxin revealed that the side chain of AE can form strong interactions with Lys110, Tyr112, and Met113. As shown in Figure 3B, the complex was found to reach equilibrium at 50 ns based on the analysis of the root-mean-square deviations (RMSD) of backbone C_α_ atoms, which indicated that the complex system reached the equilibrium.

To explore the energy contributions from residues of the binding sites in the α-toxin-AE complex, we calculated the energy decomposition for the α-toxin-AE complex system. As shown in Figure 3C, Tyr112 makes a strong contribution to the total binding energy, with an Δ*E*_*total*_ ≤ −2.0 kcal/mol. In addition, the Lys110 and Met113 residues also have appreciable total binding energy contributions, with Δ*E*_*total*_ values of ≤ −0.5 kcal/mol. The results suggest that these three residues are key residues for AE.

**Figure 4:**
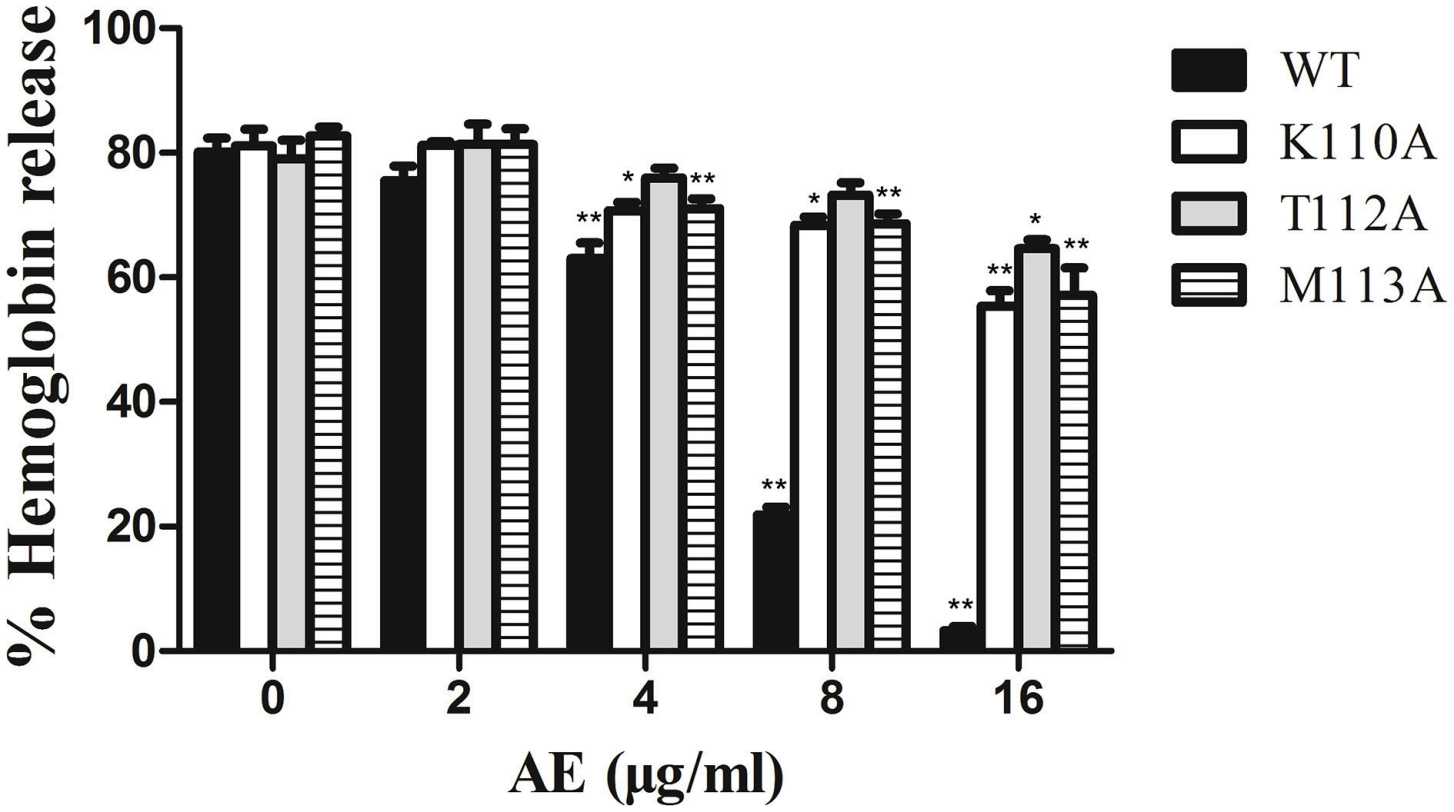
The effect of AE on the hemolytic activity of α-toxin and its three mutants. Recombinant α-toxin was preincubated with various concentrations of AE and the hemolytic activity of toxin was determined using hemolysis assay as described in Figure 1E. Bars represent the mean values of the experiments (^*^ indicates *P* < 0.05 and ^**^ indicates *P* < 0.01 compared with the AE-free group; two-tailed Student's *t*-test).

To confirm these theoretical results, we calculated the total binding free energy for the α-toxin-AE complex and their detailed energy contributions according to the MM-PBSA approach, which are summarized in Table 2. According to the calculation results, the binding free energy, Δ*G*_*bind*_, of the interaction between AE and the protein decreased in the following order: WT-α-toxin > mutants, indicating that the WT-α-toxin had the strongest AE-binding ability. By fluorescence spectroscopy quenching, we measured the Δ*G*_*bind*_ and the number of binding sites between AE and the three mutants, and these results were consistent with those obtained by computational methods (Table 2). Another hemolysis assay was used to determine the effect of AE on the hemolytic activity of the mutant proteins. As expected, the sensitivity of these mutants to AE was much lower than that of the WT protein, indicating that the mutations affected the binding of AE to α-toxin (Figure 4).

**Table 2:** The binding free energy (kcal/mol) of the WT-α-toxin-AE, K110A-AE, Y112A-AE, and M113A-AE systems based on computational analysis and the values of the binding constants (*K*_*A*_) based on fluorescence spectroscopy quenching.

	**WT-α-toxin**	**K110A**	**Y112A**	**M113A**
Computational method	−12.6 ± 1.1	−8.2 ± 0.8	−7.7 ± 1.0	−8.1 ± 0.9
*K*_A_ (1 × 10^4^) L·mol^−1^	8.9 ± 0.8	5.8 ± 0.6	6.1 ± 0.9	7.1 ± 0.7

Taken together, these results indicate that the molecular dynamics (MD) simulation of the α-toxin-AE complex is reliable and that binding of the AE inhibitor at the active site (residues Lys110, Tyr112, and Met113) of α-toxin leads to inhibition of its activity.

### AE Protects A549 Cells From USA300-mediated Cell Injury

**Figure 5:**
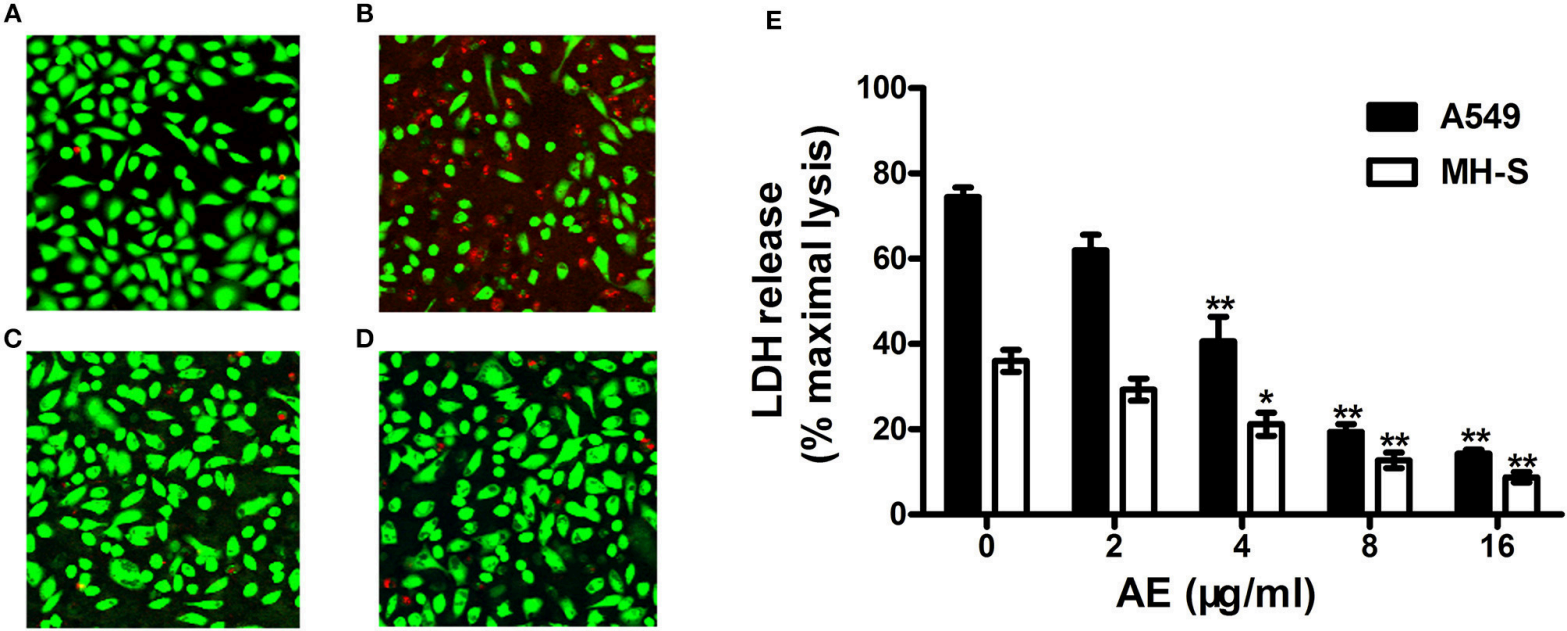
AE alleviates the injury of cells caused by USA300. *S. aures* infected A549 cells were treated with or without AE and stained with live (green)/dead (red) reagents. Then, the cells were detected with a confocal laser scanning microscope. **(A)** Uninfected A549 cells; **(B)** cells infected with USA300 in the absence or **(C)** presence of 16 μl/ml of AE; **(D)** cells cocultured with 16 μl/ml AE. **(E)** Cells were infected with *S. aureus* at an MOI of 500 for 5 h at 37°C and the LDH release by A549 cells and MH-S cells with or without AE was evaluated using a Cytotoxicity Detection Kit. Bars represent the mean values of the experiments (^*^ indicates *P* < 0.05 and ^**^ indicates *P* < 0.01 compared with the AE-free group).

Live/dead cell staining and lactate dehydrogenase (LDH) release assays were performed to assess the protective effects of AE on *S. aureus*-mediated A549 cell injury in the coculture system of *S. aureus* and cells with or without AE. After incubation for 5 h, the cells were stained with live/dead (green/red) reagent (Figure 5A). As shown in Figure 5B, most of the cells were dead (red fluorescence) in the control group without AE. However, the cell injury was decreased with increasing concentrations of AE, and almost no dead cells could be observed under a confocal laser scanning microscope when the concentration of AE reached 16 μg/ml (green fluorescence for live cells, Figure 5C). Furthermore, the cells treated with 16 μg/ml of AE only showed a normal cell morphology, with almost no dead cells (Figure 5D), suggesting that no toxicity could be observed at therapeutic concentrations.

LDH can be released into culture medium as an indicator of cell lysis. Thus, LDH release assays of A549 cells and mouse lung macrophages (MH-S) were used to quantify cell death. As shown in Figure 5E, ~74.4% of A549 cells and 36.1% of MH-S cells were dead after incubation with USA300 for 5 h without AE. Significantly, when AE was added at 16 μg/ml, the rate of cell death was decreased to 14.31 and 8.75%, respectively (Figure 5E). Taken together, the results indicated that AE protected against cell injury mediated by α-toxin in both A549 cells and MH-S cells *in vitro*.

### AE Protects Mice From *S. aureus* Pneumonia

**Figure 6:**
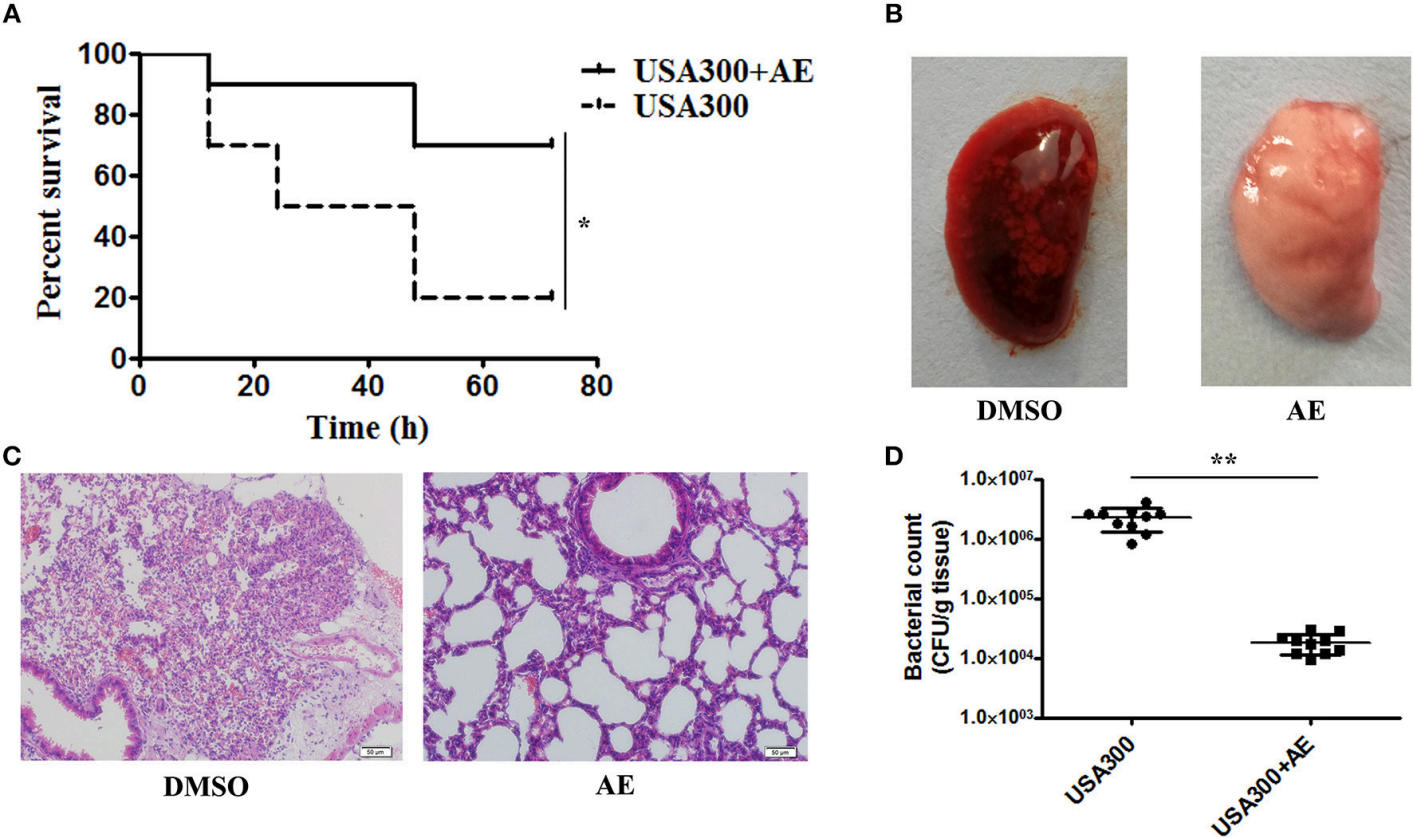
AE protects mice from USA300 pneumonia. C57BL/6J female mice were administered nasal drops with 20 μl of suspended bacteria (1 × 10^10^ CFUs/mL) and treated with or without 100 mg/kg AE by hypodermic injection. **(A)** The mortality of infected mice treated with AE or DMSO. Log-rank test was used for the estimation of the mortality rate at 72 h. **(B)** The pathological changes observed by the naked eye and **(C)** a light microscope. **(D)** The effects of AE on the colonization of USA300 in the lungs (^*^ indicates *P* < 0.05 and ^**^ indicates *P* < 0.01 compared with the AE-free group).

Following the determination of the protective effect of AE against *S. aureus* virulence *in vitro*, we further investigated whether this effect occurred *in vivo* by establishing a mouse model of *S. aureus* pneumonia. C57BL/6J mice were infected with USA300 as previously described (Qiu et al., 2012a,b) and treated with AE or DMSO for 72 h. The mortality of infected mice that received AE was significantly decreased compared with that of mice injected with DMSO (Figure 6A). The lungs of the mice were pathologically analyzed. The redness of the lungs from the AE-treated group was much lighter than that of the control group when observed by the naked eye (Figure 6B), and the alveolar space was obliterated by inflammatory cell infiltrates in the control group as shown by microscopy (Figure 6C). Furthermore, we quantified the colonization of bacteria in the lungs. As shown in Figure 6D, the quantity of bacteria in lungs treated with AE was much lower than that of the control group. Taken together, our results indicated that AE is an effective candidate for protecting mice from *S. aureus* pneumonia.

## Discussion

Over the past 100 years, the application of antibiotics has saved countless lives, and scholars once believed that human beings could completely eradicate diseases caused by bacteria. However, the abuse of antibiotics has gradually increased resistance, which has finally led to the elimination of antibiotics after decades of application and has resulted in many multidrug resistant strains that are insensitive to conventional antibacterial strategies, such as MRSA (Rasko and Sperandio, 2010). The use of antibiotics is typically related to the survival of bacteria and always leads to the selection of resistant strains that gradually become dominant and eventually results in resistant flora. Thus, it is possible that most antibiotics may eventually induce drug resistance due to the excellent ability of bacteria to adapt to the environment (Cegelski et al., 2008). Therefore, new treatment strategies are needed that are less effective at selecting for resistance. In this study, we observed that AE did not affect the viability of *S. aureus* at the concentrations tested (2–16 μg/ml). These results indicate that AE would not put selective pressure on *S. aureus*.

In recent years, many studies have suggested the targeting virulence factors as an alternative strategy and have shown some impressive results (Qiu et al., 2012a,b). For *S. aureus* infections, many virulence factors are employed during the invasion, and most of them, such as α-toxin, are not correlated with bacterial survival. Based on previous studies, the destruction of the air-blood barrier by α-toxin is one of the most important factors in *S. aureus* pneumonia (McElroy et al., 1999; Xu et al., 2015). Accordingly, in this study, we attempted to treat *S. aureus*-induced pneumonia by targeting α-toxin. We identified AE as a potential inhibitor of α-toxin, as it directly interferes with the hemolytic activity of α-toxin without affecting the production of α-toxin by *S. aureus* or bacterial viability. By means of MD simulation, we found that AE could localize to the catalytic pocket of α-toxin (residue 100–150), which is very close to the binding site of substrate, by directly engaging residues K110, T112, and M113. These interactions blocked the binding of α-toxin and its substrate and finally led to a loss of biological activity of α-toxin. Furthermore, we also demonstrated that AE had protective effects in a tissue infection model and animal model of *S. aureus* pneumonia. These results indicated that AE is a potential treatment for *S. aureus* infections, especially MRSA infections. In addition, our results confirmed the feasibility of the anti-virulence strategy.

Interestingly, *Aloe vera, Rheum officinale*, and semen cassiae torae, which are the major sources of AE, have been used as traditional Chinese medicines in the treatment of lung infections for thousands of years. Although this study cannot show that the effect of AE on α-toxin is the major mechanism of these medicines on lung infections, it provides support for the theory of traditional Chinese medicine at the molecular level.

Previous studies have reported that α-toxin can also damage the membranes of B cells and T cells, which may lead to an attenuated immune response and create a stable and lasting bacterial colonization environment (Nygaard et al., 2012). As reported in several studies, subinhibitory concentrations of beta-lactam antibiotics may increase α-toxin expression, indicating that infections may be uncontrollable when treated with beta-lactams (Ohlsen et al., 1998). Therefore, targeting α-toxin by AE may support the therapeutic effects of immune cells and antibiotics, but this strategy requires further confirmation. Finally, our results indicated that AE is a promising candidate for *S. aureus* infection by targeting α-toxin.

## Data Availability

The raw data supporting the conclusions of this manuscript will be made available by the authors, without undue reservation, to any qualified researcher.

## Ethics Statement

Six-to-eight-week-old C57BL/6J female mice were purchased from Jilin University Experimental Animal Center and were handled according to the standards approved by the Animal Welfare and Research Ethics Committee of Jilin University.

## Author Contributions

ZT, JW, and LJ conceived and designed the experiments. LJ, TY, and ZS performed the experiments. LJ analyzed the data. ZT and JW wrote the paper.

### Conflict of Interest Statement

The authors declare that the research was conducted in the absence of any commercial or financial relationships that could be construed as a potential conflict of interest.
